# The Prevalence of Acceptance Between General Anesthesia and Spinal Anesthesia Among Pregnant Women Undergoing Elective Caesarean Sections in Saudi Arabia

**DOI:** 10.7759/cureus.44972

**Published:** 2023-09-10

**Authors:** Nasser A Tawfeeq, Faisal Hilal, Noof M Alharbi, Fay Alowid, Rana Y Almaghrabi, Rahaf Alsubhi, Shahd F Alharbi, Amal Fallatah, Leenah M Aloufi, Noor A Alsaleh

**Affiliations:** 1 Department of Anesthesiology, King Abdulaziz Medical City in Riyadh (KAMC-RD), Riyadh, SAU; 2 Department of Anesthesiology and Pain Management, King Abdullah Medical Complex - Jeddah (KAMCJ), Jeddah, SAU; 3 College of Medicine, King Saud bin Abdulaziz University for Health Sciences, Riyadh, SAU; 4 College of Medicine, Ibn Sina National College for Medical Studies, Jeddah, SAU; 5 College of Medicine, Qassim University, Buraydah, SAU; 6 College of Medicine, Al-Rayan Colleges, Al-Madinah, SAU; 7 College of Medicine, Al-Rayan Colleges, Al Madinah, SAU; 8 College of Medicine, King Faisal University, Al-Ahsa, SAU

**Keywords:** delivery, elective caesarean section, general anesthesia, spinal anesthesia, labor, c-section, cesarean

## Abstract

Background

The choice of anesthesia for an elective cesarean section should be based on an individual benefit-risk assessment, considering the pregnant woman’s preferences, concerns, and the available medical expertise. This study aimed to determine the preferences for general and spinal anesthesia among women undergoing elective cesarean sections and the factors affecting their choice.

Methods

The study design is a cross-sectional study, and it was conducted on pregnant women to measure the acceptance of general anesthesia and spinal anesthesia in patients with elective cesarean sections in Saudi Arabia. Random pregnant women were invited to participate in this study across Saudi Arabia after fulfilling the inclusion criteria. A digital questionnaire was distributed across Saudi Arabia to be filled out by female residents. A Microsoft Excel (Microsoft Corporation, Redmond, Washington, USA*)* sheet was used for data entry, while IBM SPSS software version 27.0.1 (IBM Corp., Armonk, New York, USA) was used for statistical analysis.

Results

The study included 813 participants; most (28%) of them were 25-30 years old. Of the study participants, 54% had chosen spinal anesthesia before, 22% had chosen general anesthesia, and 24% had chosen neither. Reasons to choose general anesthesia were reported as follows: 21.6% feared pain during surgery, 24.2% feared watching the surgical procedures on their bodies, 16.6% feared back pain, 12.8% feared being paralyzed, and 15.1% feared needles used to administer anesthesia in the lower back. Reasons for choosing spinal anesthesia were reported as follows: 26.3% had back pain concerns; 13% feared prolonged unconsciousness; 9.6% feared having a headache after surgery; 17% had post-surgery pain concerns; 30.1% wanted to be alert at the time of the birth of the baby; 10.6% feared the chances of experiencing nausea and vomiting; and 7.4% feared not being able to breastfeed.

Conclusion

Spinal anesthesia was chosen by more participants than general anesthesia. There was a statistically significant association between choosing spinal anesthesia and the number of previous pregnancies, parity, history of preterm labor, and recommendation to undergo general or spinal anesthesia by non-medical staff. It was also significant with the older age and higher educational level of participants. This decision may be influenced by a number of variables, the most significant of which are prior experience with general anesthesia or spinal anesthesia, educational attainment, and non-medical advice.

## Introduction

An elective cesarean section is a common surgical procedure performed on pregnant women, and the choice of anesthesia is an important consideration. In Saudi Arabia, both general anesthesia (GA) and spinal anesthesia (SA) are used for elective cesarean sections, with each being chosen based on its safety and ability to help both the mother and fetus, but acceptance of these techniques among pregnant women varies [1].

Studies show that pregnant women who choose to undergo elective cesarean sections prefer general anesthesia to spinal anesthesia for a number of concerns including watching the surgical procedures on their bodies, the risk of back pain after surgery, and spine damage concerns [2,3]. However, the most prevalent concerns among the women who choose spinal anesthesia over general anesthesia are a desire to be awake throughout childbirth to see the baby and a fear of prolonged unconsciousness should they choose general anesthesia [2].

General anesthesia uses intravenous drugs or inhalers to induce unconsciousness, whereas spinal anesthesia involves injecting an anesthetic into the cerebrospinal fluid in the lower section of the spine to induce numbness by blocking nerve signals [1]. Clinically, the choice of anesthesia for elective cesarean delivery depends on several factors, including maternal and fetal health, fetal gestational age, and the preferences of the pregnant woman and obstetrician [4]. It is therefore crucial that a woman be adequately informed about each anesthetic procedure, expected results, and any potential adverse effects beforehand so as to make an informed choice.

Improving pregnant women’s understanding and attitude about anesthesia as part of regular preoperative patient education is crucial for a successful perioperative result, managing medico-legal situations, and patients’ decision-making. Adequate patient knowledge and a positive attitude toward anesthesia have been shown to improve the patient’s results after cesarean delivery [5].

Spinal or regional anesthesia has been favored as the best option for elective, uncomplicated cesarean deliveries thanks to its avoidance of the patient’s respiratory path, decreased risk of aspiration of gastric contents, and ease of use [6]. However, general anesthesia is still administered, particularly when regional anesthesia is inefficient or contraindicated.

The prevalence of acceptance between GA and SA in pregnant women undergoing elective cesarean sections in Saudi Arabia has been investigated in several research studies [7-9]. Most studies have reported higher acceptance rates for SA compared to GA, citing fewer complications, faster recovery times, and a lower risk of maternal and fetal morbidity and mortality [7,8]. However, other studies have reported higher acceptance rates for GA, with women citing fear and anxiety associated with spinal procedures and concerns about awareness and pain during surgery [9,10].

The choice of anesthesia for an elective cesarean section should therefore be based on an individual benefit-risk assessment, taking into account the pregnant woman’s preferences and concerns and the available medical expertise. The primary purpose of this research is to investigate the factors influencing the choice between spinal anesthesia and general anesthesia for elective cesarean section deliveries, with a specific focus on pregnant women's preferences and motivations. The study also aims to evaluate the knowledge and experiences of childbearing women regarding the type of anesthesia used during childbirth. The motivation behind this research is to gain a better understanding of why some women opt for spinal anesthesia while others choose general anesthesia for cesarean deliveries. This information is important for healthcare professionals to provide appropriate guidance and support to pregnant women in making informed decisions about their anesthesia options. Additionally, the study seeks to identify any associations between demographic factors, previous experiences, and the choice of anesthesia, which can contribute to improving the quality of care and patient satisfaction in obstetric anesthesia.

## Materials and methods

Study design

A cross-sectional, questionnaire-based study design was conducted in Saudi Arabia among pregnant women from September 2022 to August 2023. The respondents (pregnant women) were randomly invited to participate by filling out a questionnaire to measure the prevalence of acceptance between general anesthesia and spinal anesthesia in patients with elective cesarean sections in Saudi Arabia.

Study participants

The participants in the study were pregnant women between the ages of 18 and 45 who lived in Saudi Arabia and were already scheduled for elective cesarean sections. The study excluded patients with chronic back pain, hypertension, diabetes, and coronary heart disease.

Study sample

The sample size was determined using the Raosoft software (Raosoft Inc., Seattle, Washington, USA), preset to a 95% confidence level and a 5% confidence interval (CI). The sample size of the study was 813 participants, pregnant women between the ages of 18 and 45, across different regions of Saudi Arabia.

Sampling technique

A convenient non-probability sampling technique was used to obtain a representative sample of the targeted population. This sampling technique was adopted due to its ease of data collection and reaching the target population.

Inclusion and exclusion criteria

A digital questionnaire was distributed across Saudi Arabia to be filled by female residents who fulfilled the following criteria: female residents aged 18-45 years, pregnant women who are scheduled for an elective cesarean section and going for either spinal or general anesthesia, and females who had undergone elective cesarean sections under spinal or general anesthesia.

The study excluded females under 18 and over 45 years of age, pregnant women who are going for normal vaginal delivery, and pregnant women undergoing emergency cesarean sections.

Study setting

The study survey was distributed in Arabic and English via social media in the form of an online Google form. The data were analyzed using the IBM SPSS software version 27.0.1( IBM Corp., Armonk, New York, USA). The research was reviewed and approved by the King Abdullah International Medical Research Center (KAIMRC), Riyadh, Saudi Arabia.

Data collection

This cross-sectional study was conducted through an online Google Forms (Google LLC, Mountain View, California, USA) survey. Data collectors were assigned to collect the data after obtaining ethical approval (numbered IRB/1611/23 and study number NRC23R/372/06 from KAIMRC). Ethical consent to collect personal data was obtained from pregnant women electronically; the goal of the study was explained, and they were informed that participation is voluntary and not compulsory; they have the right to withdraw from the study at any time; and the participants can ask any question related to the study. All the data were collected and processed for the study only. The confidentiality and anonymity of participants were upheld to protect their privacy. The data collection process was done by distributing an online Google Forms survey between July 2023 and August 2023. Data were collected using a questionnaire that only investigators had access to. The collected data were automatically linked to a Spreadsheet file, a standard feature in Google Forms.

Statistical analysis

All data were entered in Microsoft Office Excel (Microsoft Corporation, Redmond, Washington, USA) and then transferred to IBM SPSS software. All statistical analyses were executed using IBM SPSS version 27.0.1. Descriptive statistics of the participants’ sociodemographic characteristics were expressed in the form of frequencies and percentages. Quantitative data were presented as the mean and standard deviation. Qualitative variables were summarized using frequencies and percentages. A chi-square test was used to test associations between categorical variables. A P-value of < 0.05 was considered statistical significance with a 95% confidence level at 5% CI.

## Results

Demographics

The study included 813 participants, of whom 28% were aged between 25-30 years, 23% were 31-35 years old, and 23.4% were more than 40 years old. Most (38.6%) of the participants were from the western region of Saudi Arabia, while the northern region had the least (5.2%) participants; 91.4% of participants lived in cities and 8.6% lived in villages; 30.9% of participants worked in government jobs, 16.4% in the private sector, and 43.9% were housewives. Regarding educational level, 59.2% had a bachelor’s degree, 19.7% had a high school degree, and 13.7% had a diploma (Table 1).

**Table 1:** The sociodemographic characteristics of participants (n=813) Frequencies, percentages, and Chi-square test

Sociodemographic variables		No.	Percentage	P-value
Age (in years)	less than 25	49	6.0%	0.001*
	25 - 30	228	28.0%	
	31 - 35	187	23.0%	
	36 - 40	159	19.6%	
	More than 40	190	23.4%	
Region	Central region	232	28.5%	0.901
	Eastern region	154	18.9%	
	Northern region	42	5.2%	
	Southern region	71	8.7%	
	Western region	314	38.6%	
Place of residence	City	743	91.4%	0.674
	Village	70	8.6%	
Occupation	Government job	251	30.9%	0.016*
	Private sector job	133	16.4%	
	Self-employed	34	4.2%	
	Student	38	4.7%	
	Housewife	357	43.9%	
Level of education	High school	160	19.7%	0.008*
	Bachelor's degree	481	59.2%	
	Diploma	111	13.7%	
	Master's degree or higher	61	7.5%	

Associated factors

This study sought to identify multi-variant factors associated with choice of anesthesia, where 20.3% of participants reported one pregnancy, 24% two pregnancies, and 23.4% fifth or more. As for parity, 23.7% reported one, 17.2% reported two, and 31% reported four or more. Additionally, 33.3% reported a history of preterm labor; 43.4% of participants reported that they had been recommended by non-medical staff to undergo spinal anesthesia; 10.5% had been recommended to undergo general anesthesia; and 46.1% received no recommendations at all.

Table 2 below shows a statistically significant association between choosing general or spinal anesthesia and the number of previous pregnancies, parity, history of preterm labor, and recommendation to undergo general or spinal anesthesia by non-medical staff.

**Table 2:** Participants' determinants and their association with choosing general or spinal anesthesia (n = 813). Frequencies, percentages, and chi-square test

Parameters		No.	%	P-value
Number of previous pregnancies	First pregnancy	165	20.3%	0.001*
	Second pregnancy	195	24.0%	
	Third pregnancy	134	16.5%	
	Fourth pregnancy	129	15.9%	
	Fifth or more	190	23.4%	
Parity	Never	107	13.2%	0.001*
	Once	193	23.7%	
	Twice	140	17.2%	
	Three times	121	14.9%	
	Four or more	252	31.0%	
History of preterm labor	Yes	271	33.3%	0.001*
	No	542	66.7%	
Recommended to undergo general or spinal anesthesia by non-medical staff	No recommendations by non-medical staff	375	46.1%	0.001*
	Spinal anesthesia	353	43.4%	
	General anesthesia	85	10.5%	

It was also significant with the older age and higher educational level of participants (Table 1).

History of anesthesia

**Figure 1:**
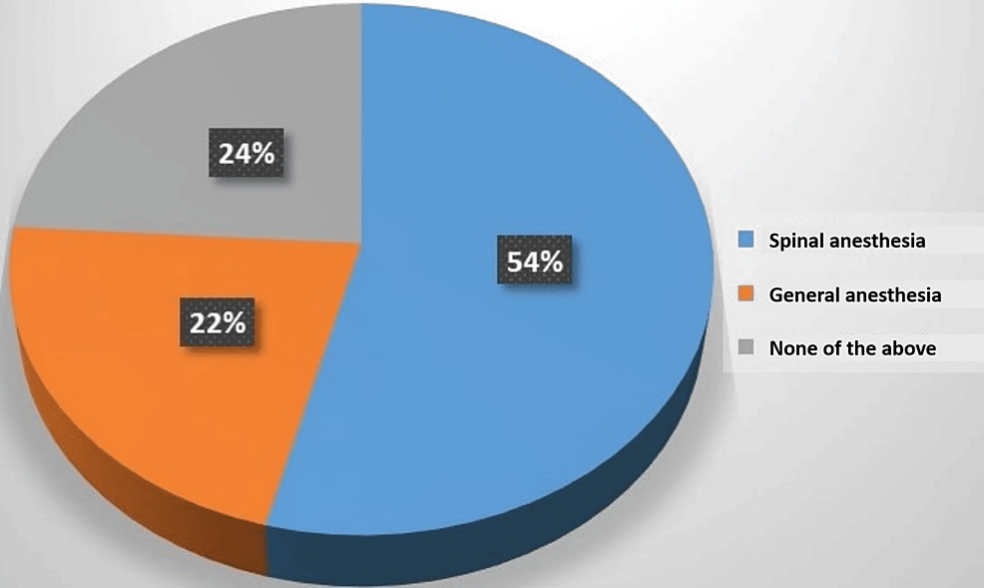
Participants' previous experience with general or spinal anesthesia (n= 813)

As illustrated in Figure 1, 54% of study participants had chosen spinal anesthesia before, 22% chose general anesthesia, and 24% couldn’t choose either.

Reasons for choosing general or spinal anesthesia

Table 3 illustrates reasons for choosing general or spinal anesthesia.

**Table 3:** Reasons for choosing general or spinal anesthesia (n= 813)

Parameter		No.	%
Reason to choose general anesthesia	I didn't choose general anesthesia	457	56.2%
	Fear of pain during surgery	176	21.6%
	Fear of watching the surgical procedures on my body	197	24.2%
	Fear of back pain	135	16.6%
	Fear of being paralyzed	104	12.8%
	Fear of needles in the back	123	15.1%
Reason to choose spinal anesthesia	Fear of pain	214	26.3%
	Fear of not waking up	106	13.0%
	Fear of headache	78	9.6%
	Fear of pain after surgery	138	17.0%
	The desire to be alert at the time of the birth of the baby	245	30.1%
	Fear of nausea and vomiting	86	10.6%
	Fear of urinary retention	69	8.5%
	Fear of not being able to breastfeed	60	7.4%
	I didn't choose spinal anesthesia	239	29.4%

Reasons for general anesthesia were reported as follows: fear of pain during surgery (21.6%), fear of watching the surgical procedures on their bodies (24.2%), risk of back pain after surgery (16.6%), fear of being paralyzed (12.8%), and fear of needles in the back (15.1%). Reasons to choose spinal anesthesia were as follows: fear of pain (26.3%), fear of not waking up (13%), fear of headache (9.6%), fear of pain after surgery (17%), desire to be alert at the time of the birth of the baby (31.1%), fear of nausea and vomiting (10.6%), fear of urinary retention (8.5%), and fear of not being able to breastfeed (7.4%).

## Discussion

A primary cesarean delivery is referred to as a cesarean delivery on maternal request (CDMR) if the woman requests this type of delivery rather than the usual medical or obstetrical considerations. Less than 3% of all cesarean births worldwide and 1%-18% in the United States had CDMR [11, 12]. The study also aimed to investigate the acceptance of the type of anesthesia among pregnant women with elective cesarean sections and evaluate the knowledge that childbearing women have about the type of anesthesia.

Neuraxial anesthesia is the preferred anesthetic approach for cesarean deliveries in most nations unless there is a complication [13]. This is based in part on the population’s higher death and morbidity rates following general anesthesia. The estimated case fatality rate for general anesthesia during cesarean birth between 1991 and 1996 was 16.8 deaths per million of general anesthetics administered; this fell to 6.5 deaths per million between 1997 and 2002 when general anesthetics were administered. On the other hand, deaths resulting from regional anesthetics dropped from 2.5 deaths per million anesthetics to 3.8 deaths per million anesthetics in the same period. However, the predicted case fatality rate of regional anesthesia during cesarean birth increased slightly [13]. General anesthesia is therefore recommended for cesarean deliveries in addition to emergency conditions (35%) and the woman’s refusal to have spinal anesthesia (20%) [14].

Spinal anesthesia has been established in prior research to enhance clinical results and reduce cesarean birth complications, although the health-related quality of life (HRQoL) has not been previously examined. To investigate the impact of regional anesthesia versus general anesthesia on the results of cesarean delivery, Afolabi and Lesi conducted a systematic analysis of 20 publications and included 1793 women who underwent cesarean delivery in 2012; the evidence from the analysis was insufficient to conclude that regional anesthesia was preferable to general anesthesia [15]. As demonstrated by Gursoy et al., neuraxial anesthesia permits patients to resume regular daily activities sooner than general anesthesia. Additionally, when regional anesthesia was used instead of general anesthesia 24 hours after cesarean delivery, the EuroQol- 5 Dimension (EQ-5D) general health score was higher [16, 17].

According to our study results, 54% of study participants had chosen spinal anesthesia before, 22% chose general anesthesia, and 24% couldn’t or didn’t choose either beforehand. In a previous study, 40% of participants chose spinal anesthesia, whereas 60% chose general anesthesia. The most frequently cited reasons against choosing spinal anesthesia were concerns over spinal cord injury (64.3%) and discomfort from sight and sound during surgery (53.3%), while concerns about not waking up during general anesthesia (54.3%) and a desire to be awake during childbirth (40.7%) were most frequently cited against choosing general anesthesia.

According to research by Sadeghi et al., 50% of women in Tehran chose general anesthesia, 30% chose spinal anesthesia, and 20% didn't make a decision [18, 19, 20]. General anesthesia was the preferred option in several studies carried out in Iran, including those carried out at Shahrekord University Hospital, where 64% of women chose general anesthesia, and two hospitals in Torbate Heidayyeh, where 100% of C-sections were carried out under general anesthesia [21]. Additionally, many patients (70%) in the study by Bukar et al. in Nigeria selected general anesthesia [22].

The participants' reasons for choosing general anesthesia were: 21.6% said it was due to fear of pain during surgery; 24.2% said it was due to discomfort from sight and sound during surgery in the operating room; 16.6% said it was fear of back pain; 12.8% said it was fear of being paralyzed; and 15.1% said it was fear of needles in the back. Reasons to choose spinal anesthesia were reported as follows: 26.3% said it was due to fear of pain, 13% said it was the fear of not waking up, 9.6% said it was due to fear of headache, 17% said it was due to fear of post-surgery pain, 30.1% said it was because of the desire to be alert at the time of the birth of the baby, 10.6% said it was due to fear of nausea and vomiting, and 7.4% said it was due to the fear of not being able to breastfeed. This was comparable to the results of previous studies: the most common justifications for selecting general over local anesthesia were anxiety about back pain, fear of a spinal cord injury, and concerns about discomfort from sight and sound during surgery. The fear of not waking up and the desire to remain awake during childbirth were among the most prevalent justifications for selecting local anesthesia [18].

According to a study by Bukar et al., fear of pain, injury, and prolonged unconsciousness were the most frequent justifications for selecting general anesthesia, while the most frequent justification for choosing spinal anesthesia was to witness childbirth in the operating room [22]. According to Fassoulaki et al., watching the child being delivered was the most significant decision among the women who chose spinal anesthesia, whereas, among the women who selected general anesthesia, fear of spine injury was the most significant factor. Most studies have found that fear of back pain and needle discomfort were the main deterrents to selecting spinal anesthesia due to a high level of needle phobia. The procedure for administering spinal anesthesia is characterized by the insertion of a needle into the lumbar area and is a source of concern for most patients, although this can be addressed by giving patients adequate explanations and assurance. Studies show that women who exhibit higher levels of preoperative anxiety are more likely to choose general anesthesia.

According to our study results, there was a significant association between choosing spinal anesthesia and the number of previous pregnancies, parity, history of preterm labor, and recommendation to undergo general or spinal anesthesia by non-medical staff. It was also significant with the older age and higher educational level of participants. A prior study found no correlation between a history and the choice of general anesthesia. However, there was a substantial correlation in the case of spinal anesthesia. This showed that women who had experienced spinal anesthesia directly were more inclined to select it once more [23].

A different study found that women who opted for spinal anesthesia were between the ages of 25 and 40 and wanted to be awake during delivery, while those under the age of 25 cited prolonged unconsciousness as a major cause for concern. Therefore, women having their first cesarean delivery need preoperative support when choosing their preferred mode of anesthesia administration. There was no correlation between education and the choice of anesthesia. There was a correlation between education and justification for anesthesia choice [18]. According to Jathar et al., there is a direct correlation between education level and awareness of anesthesia, which increased more following the preoperative visit to the anesthesiologist [24].

## Conclusions

Spinal anesthesia was preferred by more participants to general anesthesia. There was a significant association between choosing spinal anesthesia and the number of previous pregnancies, parity, history of preterm labor, and recommendation to undergo general or spinal anesthesia by non-medical staff. It was also significant with the older age and higher educational level of participants. This decision may be influenced by a number of variables, the most significant of which are prior experience with general anesthesia or spinal anesthesia, educational attainment, and non-medical advice. The majority of the unfounded justifications stated by moms for refusing spinal anesthesia can be diminished by empowering and educating women. It is advised to carry out more research using a larger sample size and to look at the choices made regarding the type of anesthesia both before and after training and preoperative counseling.
